# Weak Lungs

**Published:** 1876-08

**Authors:** 


### WEAK LUNGS.

We believe that with proper and timely care not one in a hundred persons, predisposed to consumption, would die of that disease. The trouble is that very few apply preventive measures soon enough, and when they do commence to care for themselves they employ certain popular remedies that do more harm than good. The various cough medicines contain opium. They frequently quiet the irritation of the throat, and for a brief season arrest the cough, leading the patient to think he is recovering.

The hope is illusive. He finds that the fires have been smothered, not quenched. Opium always debilitates and impairs the power of the system to resist and throw off disease. Besides, these cough preparations do not and can not, directly or indirectly, reach the disease, which is in the lungs and not in the throat. The only medicine that *can* reach it are tonics which act indirectly by toning up the system to a point where the disease will be thrown off. By tonics, we do not mean the various preparations of iron and quinine, exclusively. These are good in their place; but there are tonics more potent than drugs, and when wisely employed and in season will never dissappoint us. These are sunshine, fresh air exercise, and wholesome food. We have only to learn how to use them in order to overcome any hereditary tendency to the development of consumption. We have not the space in a single article to give more than the outlines of a plan of treatment, which would, doubtless, arrest nearly every case of incipient lung trouble. Hours of exposure to the light of the sun, daily, is quite as important as vigorous and regular exercise in the open air. As to foods, the range is extensive. Whatever is wholesome and nutritious, and agrees with the stomach and bowels, may be used. Cathartics should seldom be given. They always debilitate and act as a drawback to our efforts to build up the system. The " Nelaton

Suppository" will generally keep the bowels regular without inconvenience to the patient, and besides it will correct any tendency to piles. When these requisites—sunlight, air, exercise, and wholesome food—are provided, and the action of the bowels regulated, we can commence to employ another invaluable remedy; this is exercise for the lungs. The patient should go into the fresh air, throw back the shoulders and expand the lungs by inhaling as much air as is possible without inconvenience. Like other kinds of exercise, it may be overdone and result in injury; but with due caution there is nothing more essential to restoration to health.

The "Spirometer" is a most valuable instrument, but must be cautiously used by persons of delicate lungs. When we speak of its value in the treatment of lung troubles, we refer particularly to its effect on the mind of the patient. The lungs can be just as easily and just as effectually expanded and exercised without it; but the favorable impression made on the patient, as he sees that his lungs are gradually healing and expanding from day to day by the use of the instrument, is a satisfaction he should not be deprived of.

A man of medium size who can run the scale of a "Spirometer" to 240 inches, may generally go about his business. The lungs will not be likely to give him further trouble. Still, it is well occasionally to test their capacity. A person who can ward off the disease until the age of twenty-eight or thirty years, is pretty certain to escape it altogether.

As life advances, the tissues of the body become more firm, and are better able to resist disease. Finally, we repeat what we have before said, that with timely care the march of the disease can be almost always arrested.

